# Chitosan Nanofibrous Dressing Increased Angiogenesis and Anti-inflammatory Response in an Acute Wound Model in Rats: A Comparative Study

**DOI:** 10.1007/s10439-025-03842-8

**Published:** 2025-10-08

**Authors:** Yury Salkovskiy, Mahboubeh Ghanbari, Carlos P. Jara, Sara Cartwright, Pinaki Mondal, Colman Freel, Sayed Ahmadreza Razian, Jason MacTaggart, Mark A. Carlson

**Affiliations:** 1 Department of Biomechanics, University of Nebraska at Omaha, 6160 University Drive South, Omaha, NE 68182 USA; 2 Department of Surgery, University of Nebraska Medical Center, 42nd and Emile, Omaha, NE 68198 USA; 3 College of Medicine, University of Nebraska Medical Center, 42nd and Emile, Omaha, NE 68198 USA

**Keywords:** Acute wound, Chitosan, PriMatrix, Angiogenesis, Nanofibers, Rats

## Abstract

**Purpose:**

Chitosan-based materials are promising for wound healing because of their antibacterial efficacy, biocompatibility, and biodegradability, though their healing mechanisms remain unclear. This study explores the cellular and molecular mechanisms of wound healing with chitosan nanofibrous dressings.

**Methods:**

Thirty-nine Sprague Dawley rats were divided into three groups based on the type of dressings administered: (1) chitosan nanofibrous dressing + transparent film, (2) commercial scaffold + transparent film, and (3) transparent film alone (control). Full-thickness wounds (2 cm × 2 cm) were created on the dorsum, splinted, and covered with dressings. Evaluations at 7, 14, and 21 days included histological analysis, and measurements of TNF-α and iNOS levels in the wounds.

**Results:**

On day 21, epithelialization was significantly higher in the chitosan group than in the scaffold group (87.5% vs. 42.0%, *p* = 0.03). TNF-α levels were lower in both treatment groups compared to the controls. In the chitosan group, the CD68+/CD163+ ratio was lower than in the scaffold group (0.28 vs. 0.62, *p* = 0.037), and blood vessel formation was greater than in the controls.

**Conclusion:**

These results suggest that chitosan nanofibrous dressings enhance acute wound healing in rats by promoting re-epithelialization, neovasculogenesis, and maintaining low TNF-α levels in the later phases of healing.

**Supplementary Information:**

The online version contains supplementary material available at 10.1007/s10439-025-03842-8.

## Introduction

Acute wounds, accounting for 18% of the overall wound demographic [1], represent a significant health concern due to their impact on patient quality of life and the substantial financial burden they impose on healthcare systems [2]. Despite the availability of various therapeutic and surgical interventions, achieving effective and efficient healing of acute wounds continues to be a persistent challenge, necessitating advancements in treatment strategies and innovative approaches to wound care.

The biological response of the human body to cutaneous injuries is a complex physiological process orchestrated by a series of biological factors collectively working to promote healing [3]. Normally, wound healing occurs in four dynamic and overlapping stages: hemostasis, inflammation, proliferation, and remodeling [4]. However, the presence of external determinants like burns or infections, as well as intrinsic factors such as age or disease-induced pathological conditions, can impede the healing process or even stop it at the inflammatory stage. Consequently, there is a critical need for innovative approaches that promote wound healing even in the presence of negative factors [3]. The last two decades have spotlighted the potential of bioengineered constructs in facilitating rapid and uncomplicated wound healing [5]. These structures mimic connective tissue, enhancing the adherence and migration of inflammatory and tissue-remodeling cells. Interactions between cellular constituents within a wound can be modulated by the structure and composition of the bioengineered materials [6]. Chitosan is a natural cationic linear polymer derived from the deacetylation of chitin, which is a component of the arthropod exoskeletons and fungal cell walls and is the second most prevalent polysaccharide after cellulose [7]. Chitosan’s functional behavior in wound healing is influenced by its degree of deacetylation (DD) and molecular weight (MW), which affect solubility, degradation rate, and bioactivity. DD is often source dependent, with insect-derived chitosan typically showing higher DD and slower enzymatic degradation [8]. While low MW chitosan is generally linked to enhanced solubility and bioactivity, the effect of MW on wound healing remains context dependent and somewhat controversial, with studies supporting both low and high MW forms [9, 10]. Chitosan’s unique properties encompass hydrophilicity, water permeability, biocompatibility, biodegradability, and antimicrobial activity when in acidic solutions with protonated amino groups [11].

In recent decades, the beneficial effects of chitosan-based dressings on wound healing have been demonstrated in numerous preclinical studies [7, 12–15], with several progressing to clinical trials (NCT04211597, NCT02668055, NCT03907111, NCT01895933). Various forms of chitosan-based materials, such as hydrogels [15] and hydrocolloids [16], have been evaluated for their effects on the wound healing process through different mechanisms. Producing nanofibrous dressings from chitosan using advanced techniques like electrospinning and solution blowing introduces unique features, including low density, nanotopography, and a three-dimensional porous structure that mimics the extracellular matrix [17–19]. Chitosan nanofibrous dressings have demonstrated superior hemostatic properties [20], along with antioxidant and antibacterial activities, and enhanced cell proliferation [21] making them highly effective for wound treatment.

However, despite chitosan nanofibers displaying promising potential and being extensively researched, the exact cellular and molecular mechanisms underlying the healing of wounds have not been completely elucidated. In addition, a comprehensive evaluation of their wound healing efficacy compared to commercial dressings in the same condition is still pending. This lack of evaluation negatively influences the widespread adoption of chitosan nanofiber dressings in the market.

In a previous report, we presented empirical evidence from human trials that electrospun chitosan nanofibrous dressings showcased therapeutic efficacy on large-area burn injuries and donor-site wounds [22]. Hence, in this study, we aimed to elucidate the cellular and molecular mechanisms underlying the healing capabilities of chitosan nanofibrous dressings, focusing on inflammatory and angiogenesis pathways. We analyzed the histological and biological markers of wound healing in an acute full-thickness wound model in rats [23]. For comparative purposes, we employed the PriMatrix Dermal Repair Scaffold (Integra LifeSciences, USA), derived from decellularized fetal bovine dermis predominantly composed of type III collagen [24]. While numerous strategies for wound management have proven effective, PriMatrix remains one of the most frequently adopted treatments for complex skin wounds in several clinical settings over the years, offering a collagen-rich matrix to support cellular growth [24–29]. While a chitosan-based control (in the form of a film or coating) would have allowed direct comparison to isolate the benefits of the nanofibrous structure, PriMatrix provides a clinically relevant benchmark due to its frequent use in wound management. This comparison enables a broader evaluation of chitosan nanofiber dressings against a widely adopted scaffold, highlighting their relative advantages in promoting re-epithelialization and angiogenesis.

The Tegaderm transparent polyurethane film (3M, USA), devoid of adjunctive therapeutic agents, was used as a secondary dressing for wounds treated with chitosan and PriMatrix. For wounds without additional treatment, it served as the sole dressing [30].

## Materials and Methods

### Manufacturing of Chitosan Nanofibrous Dressing

Research grade chitosan (NAT-0034, 30–70 mPa·s) was purchased from Matexcel (Shirley, NY), polyethylene oxide (PEO, Mv = 5000 kDa), and acetic acid (ACS reagent) were obtained from MilliporeSigma (Burlington, MA). All reagents were used without further purification.

**Fig. 1:**
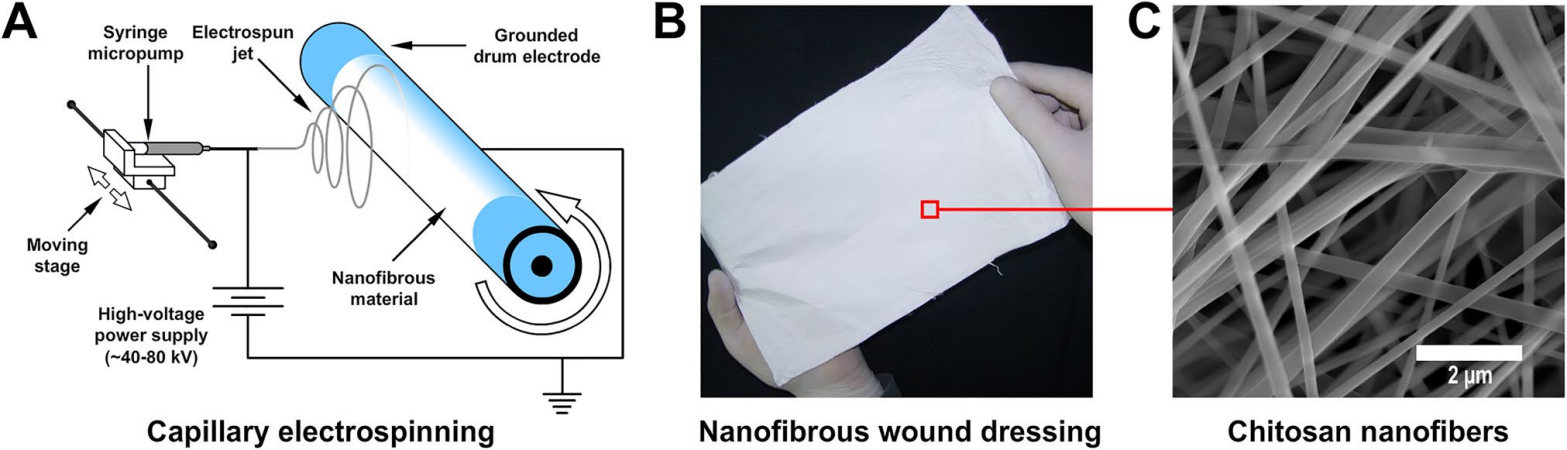
Manufacturing of chitosan nanofibrous dressing: **A** schematics of capillary electrospinning of nanofibers; **B** photo of a chitosan nanofibrous wound dressing. **C** electron micrograph close-up of the nanofibrous structure of the chitosan dressing

First, a 6% solution of chitosan in 70% acetic acid and a 1% solution of polyethylene oxide in deionized water were prepared for 24 h on a heated magnetic stirrer at 40 °C. The chitosan solution was mixed with the polyethylene oxide solution in a mass ratio of 165:10, so that the dry mass ratio of chitosan and PEO was 99:1. The resulting mixture was homogenized on a magnetic stirrer for 4 h at 40°, and then immediately used for manufacturing on our custom-made device for capillary electrospinning (Fig. 1A). Briefly, a 9 ml spinning solution was placed in a medical syringe with a 25 g blunt-tip needle which was placed horizontally in a micropump (Cole-Parmer, Model 100, USA). A high-voltage source (Spellman, SL60, USA) was connected to the syringe needle with a flexible wire, the voltage was set to 37 kV. At a distance of 180 mm from the end of the needle, a grounded steel drum collector with a diameter of 84 mm was placed, which rotated at a speed of 120 revolutions per minute.

An electric field caused a thin jet of polymer solution to emerge from the drop at the needle tip. This jet flew toward the grounded drum electrode, chaotically moved, dried, and deposited on the textile substrate as a layer of air-dry nanofibers with an average diameter of ~ 300 nm (Fig. 1B, C). The spinning solution feed rate was 4 ml/h and the total spinning time was 2.5 h. The syringe micropump was mounted on a moving linear actuator platform (GVM, GT-60D) that constantly traveled back and forth parallel to the drum for a distance of 120 mm for a period of 12 s to create a uniform thickness layer of chitosan nanofibers on the drum. The resulting chitosan nanofibrous (NF) wound dressing was separated from the drum collector and placed in a vacuum chamber for 48 h to remove acetic acid residues. To evaluate the presence of any remaining acid, the pH of distilled water (initial pH 6.32; measured with a calibrated pH meter, BANTE Instruments, China) was recorded. Chitosan NF samples (*n* = 3, 2.5 mg each) were immersed in 8 ml of distilled water in separate tubes and incubated at room temperature for 1 h. The pH of each solution was then measured to detect any leaching of acid from the samples.

Then the chitosan NF dressing was cut into squares 25 by 25 mm, packed in gas-permeable bags, and sterilized using Anprolene AN74i ethylene oxide sterilization system for 12 h. Dressings were stored in a well-ventilated room and used in animal experiments within three months of manufacture.

### Swelling and Degradation

The water absorption and biodegradation behavior of the electrospun chitosan nanofibrous mats were evaluated in vitro. Samples (*n* = 9, 1.5 cm^2^) were dried at 40 °C overnight and weighed to determine the initial dry mass (*Mi*). Each sample was immersed in 5 ml of phosphate-buffered saline (PBS, pH 7.4) and incubated at 37 °C for 1, 4, and 24 h (3 samples in each time duration). At the end of incubation, samples were removed, gently blotted with filter paper to remove excess surface liquid, and weighed to obtain the swollen mass (*M*). The samples were then re-dried at 40 °C to a constant weight (*Md*) for degradation assessment. The degrees of swelling and degradation were calculated [31] as

$$\text{Swelling }(\text{\%}) =\frac{M-Md}{Md}\times 100,$$

$$\text{Degradation }(\text{\%}) = \frac{Mi-Md}{Mi}\times 100.$$

### Animal Care and Study Design

All animal care and procedures were performed in accordance with the Institutional Animal Care and Use Committee (IACUC) policy at the University of Nebraska Medical Center. The animal study was conducted in accordance with ARRIVE guidelines [32], as detailed in the Supplementary Information. The study used 39 Sprague Dawley rats (~ 3 months old, 20 males, 19 females). To mitigate the risk of attrition related to surgical or postoperative complications, three additional rats (one per treatment group) were included in accordance with the *Guide for the Care and Use of Laboratory Animals* (NRC, 2011) [33]. The rats were housed in standard cages and maintained under controlled conditions: a 12:12 h light/dark cycle, a temperature of 23 ± 2 °C, and relative humidity at 60 ± 5%. They were provided unrestricted access to a standard diet and water for the duration of the study. Before the surgical procedure, the rats were accommodated in cages with two or three rats per cage. Following the surgery, each rat was individually housed to prevent cannibalism. The subjects were randomly allocated into three groups based on the primary dressings they received (Table 1). In each group, rats were sacrificed at intermediate (7 and 14 days) and final (21 days) time points and skin samples were harvested for histological and biological evaluations. As no animals were lost during the study, the three additional rats were retained and euthanized at the final time point (day 21), resulting in a sample size increased by one in each group.

**Table 1:** Experimental groups

Group	Primary dressing	Secondary dressing	Study group label	Number of sacrificed animals		
				Day 7	Day 14	Day 21
1	Tegaderm	–	Transparent	4	4	5
2	PriMatrix dermal repair scaffold	Tegaderm	Scaffold	4	4	5
3	Chitosan nanofibrous dressing	Tegaderm	Chitosan NF	4	4	5

### Wound Induction Procedure

Each animal was placed into the chamber for anesthesia induction with isoflurane (2–3 vol.%). Once appropriately sedated, the subject was removed from the chamber and weighed. Sustained-release Buprenorphine HCL SR LAB was administered subcutaneously into the left hind leg at a dosage of 1.0 mg/kg to provide operative and postoperative analgesia. Continuous anesthesia was administered using isoflurane via a nose cone, and adequate sedation was ensured with a toe or tail pinch. Hair was clipped circumferentially around the thorax ensuring a 5 cm × 7 cm well-shaved area on the dorsum followed by a thorough disinfection of the surgical region with three applications of scrub and a 10% povidone-iodine solution [34]. The wound perimeter (a 2 × 2 cm square area) was marked out just below the inferior borders of the scapula in the center of the dorsal surface. The subject was then draped in the standard fashion. Using a 15 mm blade, a 2 cm × 2 cm square incision was made along the marking through the skin and dermis. Using forceps, the full-thickness square was then easily elevated and removed off the back with dissecting scissors.

**Fig. 2:**
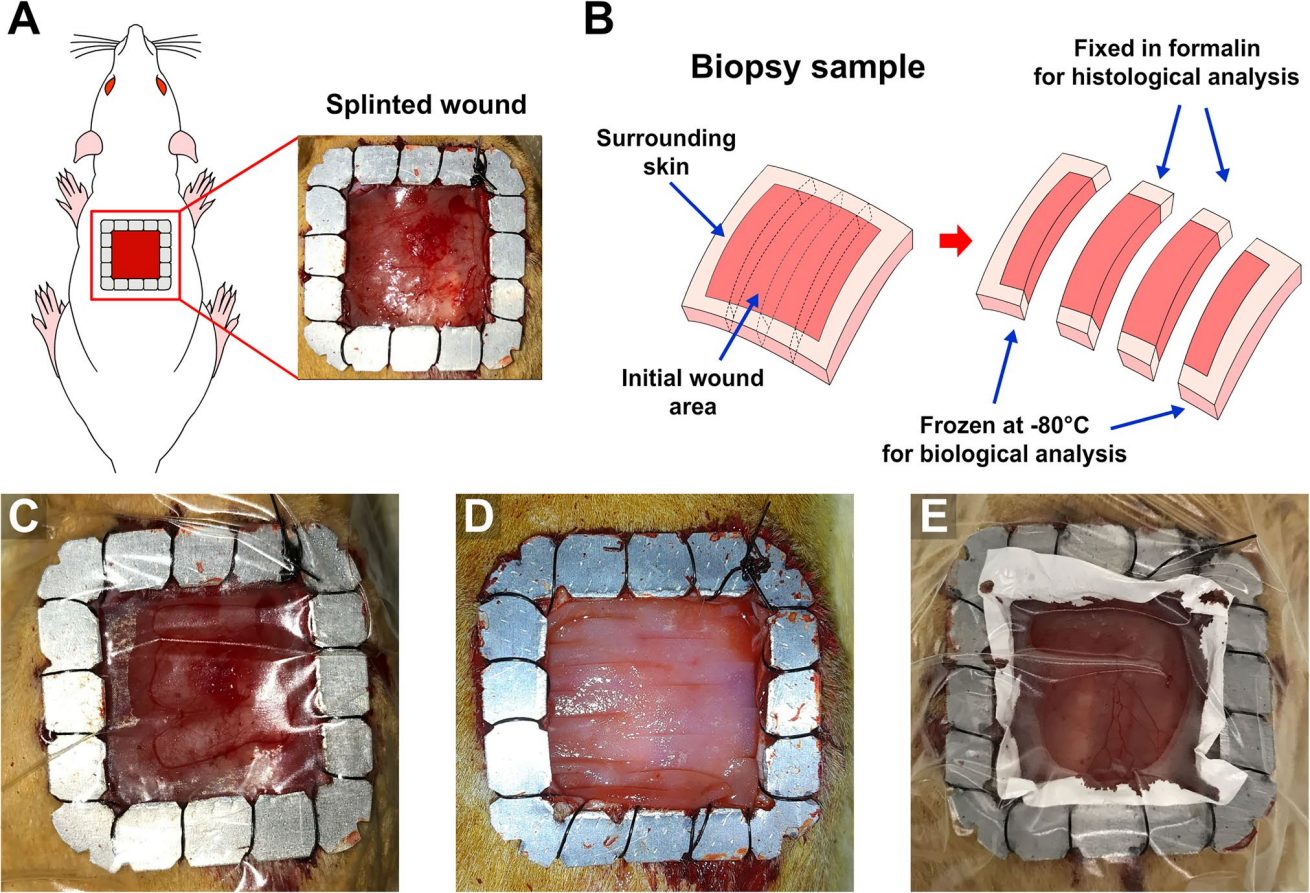
Splinted wound model: wound creation, biopsy sampling method, and dressing application. **A** Schematic illustration and representative image of a splinted full-thickness excisional wound created on the dorsal region of a rat; an aluminum splint was sutured around the wound margin to minimize contraction; **B** diagram of tissue biopsy collection, illustrating the separation of the wound and surrounding skin for both histological (formalin-fixed) and biological (frozen) analyses; **C**–**E** representative images of acute wounds treated with different dressings: **C** wound covered with a transparent dressing (Tegaderm); **D** wound covered with a PriMatrix scaffold prior to placement of the secondary dressing (Tegaderm); the scaffold matches the wound dimensions (20 × 20 mm) and fills the area of excised skin; **E** wound covered with a chitosan nanofibrous dressing applied directly to the wound bed and subsequently covered with a secondary dressing (Tegaderm); the chitosan NF dressing is larger (25 × 25 mm) than the wound to ensure complete coverage of the wound edges

**Fig. 3:**
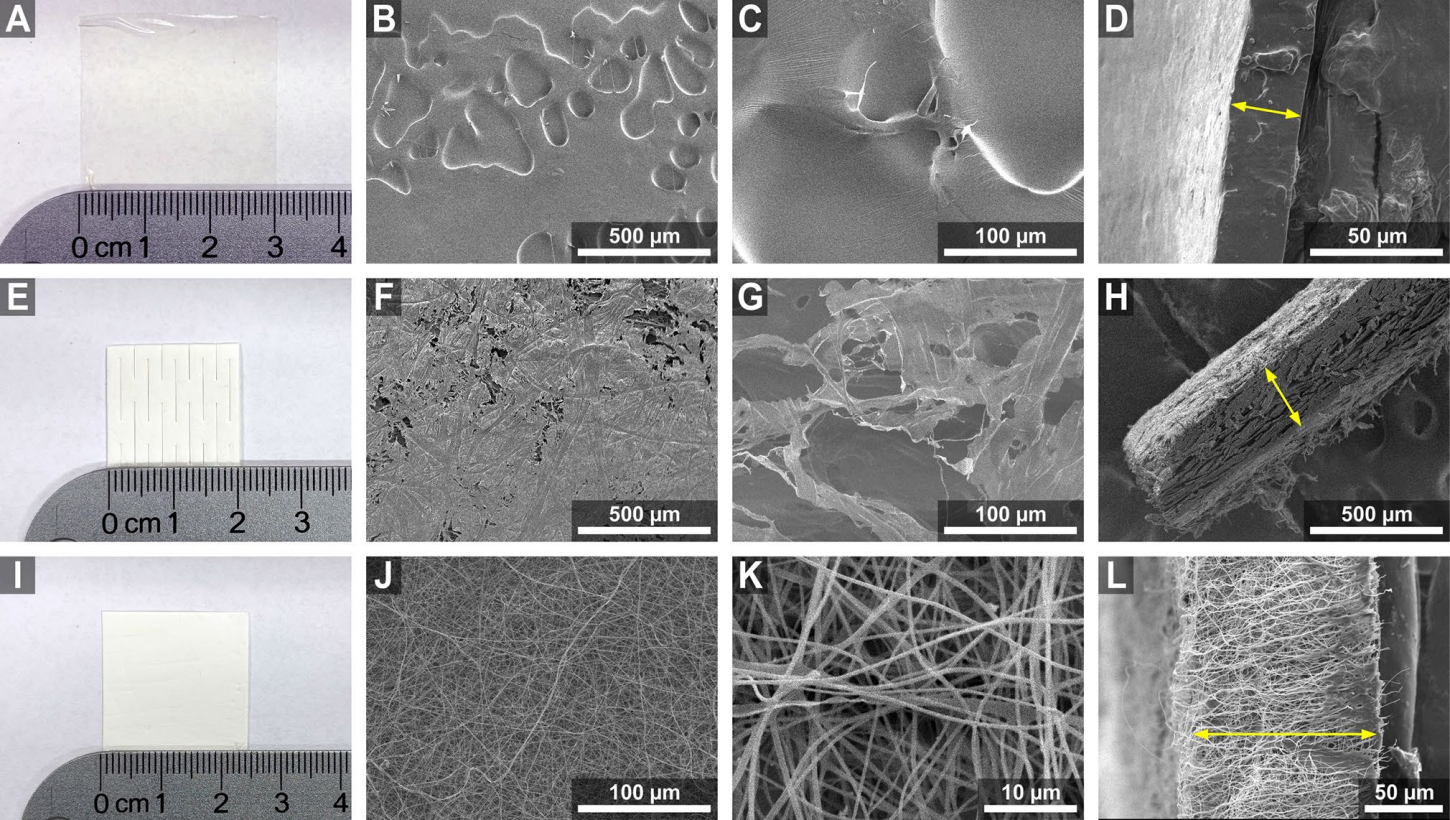
Microstructure of the wound dressings. Transparent film dressing (Tegaderm™): (**A**) photograph of the dressing; (**B**) low-magnification top view revealing shallow indentations on the film surface; (**C**) higher-magnification surface detail; (**D**) cross-sectional view highlighting the thickness (yellow arrow) and demonstrating solid, non-porous internal structure of the transparent film. PriMatrix Dermal Repair Scaffold: (**E**) photograph of the scaffold; (**F**) low-magnification top view revealing a fibrous matrix with irregular porosity; (**G**) high-magnification image of the collagen fiber architecture; (**H**) cross-sectional view indicating scaffold thickness (yellow arrow). Chitosan nanofibrous dressing: (**I**) photograph of the dressing; (**J**) low-magnification top view depicting a dense, randomly oriented network of electrospun nanofibers; (**K**) high-magnification image of continuous nanofiber morphology; (**L**) cross-sectional view illustrating the dressing thickness (yellow arrow) and internal nanofibrous structure

Since rodent wound healing primarily relies on contraction, which differs from human wounds that predominantly undergo re-epithelialization [35], we employed splinting of the wounds to inhibit contraction and simulate human-like wound healing [36–39]. A frame-shaped square splint, featuring an internal aperture measuring 2 cm by 2 cm, was sutured over the skin surrounding the wound with a non-absorbable suture to prevent wound contracture. The aluminum splint had a thickness of 0.5 mm. Small notches were created on both the external and internal boundaries of the splint to provide an even distribution of the surgical stitches on each side of the splint per splint side and facilitate a secured adherence of the wound edges to the splint (Fig. 2A). Subsequently, the splinted wounds were dressed with the corresponding group dressings. The entire wound was then covered with a transparent dressing that adhered to the surrounding skin. This transparent dressing, used as the primary dressing, covered the entire splinted wound in the same manner. Figure 2C–E presents images of each dressing applied to the dorsal region of the rats on day 0, whereas Fig. 3 illustrates the detailed microstructure of each dressing.

The thoracic region, including the wound area, was then wrapped with cotton gauze, extending from the armpits to the belly. It was secured with self-adhesive, breathable elastic tape, making sure it was snug but not so tight as to restrict breathing. The cotton gauze and elastic tape were replaced every 3 days with new ones. The primary and secondary dressings in all groups remained unchanged for the duration of the study.

### Skin Tissue Sampling and Staining

At the end of each time point (days 7, 14, and 21 post-wounding), rats of the corresponding subgroups were deeply anesthetized using CO_2_ until they reached an unconscious state, followed by euthanasia via bilateral thoracotomy. The entire wound area, along with an adjoining 5 mm of healthy skin, was excised. These skin samples were partitioned into four segments. The central two segments were immediately preserved in 10% formalin for subsequent histological assessments, while the flanking segments were rapidly stored at − 80 °C for biological evaluations, as illustrated in Fig. 2B. Post a duration of 48 h, the preserved skin tissues underwent dehydration through a graded ethanol series and were eventually embedded in paraffin. Sections with a thickness of 5 µm were sliced from these paraffin blocks. These sections, after deparaffinization, were stained using hematoxylin and eosin (H&E) (MilliporeSigma) to facilitate morphological examinations. To determine neovascularization, vimentin protein density, and the dynamics of macrophage populations, monoclonal antibodies specific for Cluster of Differentiation (CD) 31 [40], (NB100-64796, 1:50 dilution, Novus Biologicals, USA), anti-vimentin monoclonal antibody (1:200, catalog no. ab115150, Abcam, USA) [41], CD68^+^ (1:100, clone PG-M1, M0876, Agilent Dako, USA), and CD163^+^ [42] (1:200, clone, 10D6; Leica Biosystems, Buffalo Grove, IL, USA) were used, respectively.

### Histology and Immunohistochemistry Measurements

**Fig. 4:**
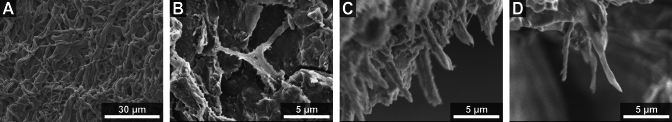
Morphological stability of chitosan nanofibrous dressing after PBS immersion and in vivo application. **A** chitosan NF dressing after 1 h of immersion in PBS at 37 °C (degradation test), where swelling-induced morphological changes are visible; **B**–**D** chitosan NF dressing after 14 days of application, with visible elements of the fibrous structure

The scanned histological images of wound tissue samples, stained with H&E and immunohistochemistry, were analyzed using a semi-automated custom-made software developed in Visual Studio C# with the .Net 4.6.2 framework and OpenCV libraries, previously utilized in several studies [43–46]. This software enables the selection of boundaries corresponding to different layers of the skin, such as the epidermis, dermis, and hypodermis, and utilizes color thresholding to assess the constituent density in each layer. Morphometric characteristics, including the average thickness and area of both the epidermis and granulation tissue (Fig. 4A and D), as well as the length of the open wound in the three groups, were measured. Additionally, the rate of epithelialization was calculated using the following formula:

## $\text{Rate of epithelialization}=\frac{(a+b)}{c}\times 100\left(\text{\%}\right)$,

Where $a$ and $b$ represent the lengths of the new epidermis at the left and right wound margins, respectively, and $c$ represents the total length of the wound [47]. These measurements were based on the optimal mathematical calculation of the selected edges by the user to ensure full coverage of the regions of interest without overlap. Additionally, the software calculated the density of new vessels (CD31 marker), vimentin filaments, and macrophages (CD68 and CD163 markers) by dividing the number of pixels selected for each component by the total number of pixels within the region of interest. These evaluations were performed in 10 randomly selected areas (3000 µm × 3000 µm) within the granulation tissue at ×20 magnification using a light microscope (Leica, USA).

### Measurement of TNF-α and iNOS Levels in Wound Tissues

The concentrations of tumor necrosis factor-alpha (TNF-α) and inducible nitric oxide synthase (iNOS) were measured in all biopsy samples taken on days 7, 14, and 21 post-wounding. First, specimens were homogenized using an ice-cold phosphate buffer solution (10 mM, pH 7.4) containing a 10 mg/ml protease inhibitor cocktail (Halt™, ThermoFisher, USA). Then, the total protein of wound specimens was determined by a BCA protein quantification kit (R&D Systems) in the homogenized tissues. The total protein in all samples was normalized to 1mg/ml. Finally, the concentrations of TNF-α and iNOS in wound tissues were measured by rat-specific ELISA kits, Abcam, USA, and MyBioSource, USA, respectively. The kits’ sensitivity for TNF-α was 25 pg/ml, and for iNOS was 0.06 ng/ml; all inter- and intra-assay CVs were < 10%.

### Statistical Analysis

Statistical analysis was performed by IBM SPSS software (Version 29). The unpaired *t*-test was used for analyzing the data on the rate of epithelialization, opening wound length, epidermal thickness, epithermal area, granulation tissue thickness, granulation tissue area, number of vessels, density of CD68^+^ and CD163^+^ markers, as well as the iNOS protein levels between the groups. One-way analysis of variance (ANOVA), followed by the Tukey HSD post hoc test, was used for analyzing data on TNF-α concentration. All data are reported as mean ± standard error of the mean (SEM), and the differences were considered statistically significant when two-tail *p*-values were < 0.05.

### Sample Size Justification

To the best of our knowledge, no prior studies have compared the efficacy of chitosan nanofibrous materials to commercial scaffolds like PriMatrix in promoting wound healing, no pre-existing data were available to guide pre-study sample size estimation. Consequently, a post hoc sample size calculation was conducted using the collected data to determine the necessary statistical power. The primary endpoint was the open wound length. Based on the observed mean differences between the chitosan NF dressing group and the transparent group, we calculated the required sample size (*N*) for each group using the formula:

$$n=\frac{{\left({z}_{1-\alpha /2}+{z}_{1-\beta }\right)}^{2}\times \left({s}_{1}^{2}+{s}_{2}^{2}\right)}{{d}^{2}},$$

where ${{d}^{2}=({\mu }_{1}-{\mu }_{2})}^{2}$ represents the squared difference between the means of the two groups, where ${\mu }_{1}$ and ${\mu }_{2}$ are the means and ${s}_{1}^{2}$ and ${s}_{2}^{2}$ are the variances (standard deviations squared) of the experimental and control groups, respectively. ${z}_{1-\alpha /2}$ is the critical value for the chosen alpha level (1.96 for $\alpha =0.05$), and and ${z}_{1-\beta }$ is the critical value for the desired power (0.84 for 80% power). The minimum number of animals required per group for each time point is as follows:

$$\text{Day }7: n=\frac{{\left(1.96+0.84\right)}^{2}\times \left({1.34}^{2}+{0.49}^{2}\right)}{{\left(14.43-11.43\right)}^{2}}=1.77,$$

$$\text{Day }14:n=\frac{{\left(1.96+0.84\right)}^{2}\times \left(3.44+{2.41}^{2}\right)}{{\left(9.08-2.54\right)}^{2}}=3.23,$$

$$\text{Day }21: n=\frac{{\left(1.96+0.84\right)}^{2}\times \left({3.05}^{2}+{3.07}^{2}\right)}{{\left(7.38-1.37\right)}^{2}}=4.06.$$

In our study, we used four animals per group for the day 7 and day 14 time points, and five animals for the day 21 time point.

## Results

### Residual Acetic Acid Evaluation by pH Measurement

The pH of the solutions after incubation with chitosan NF samples showed only minor changes compared to the initial pH of distilled water (6.32), with measured values of 6.05, 6.13, and 6.28 (mean ± SD: 6.15 ± 0.12), indicating negligible acid release from the samples.

### Swelling and Degradation

The electrospun chitosan nanofibrous mats exhibited high water uptake and minimal degradation over 24 h in PBS at 37 °C. At 1 h, the swelling ratio ranged from 553 to 814% (mean ~ 666%) with degradation between 0 and 15%, indicating good integrity. Swelling remained substantial at 4 h (518–588%, mean ~ 543%) and 24 h (456–511%, mean ~ 477%), while degradation stayed low (~ − 4% to + 5%), demonstrating stable performance over the initial wound healing period. Scanning electron microscopy revealed preservation of the fibrous structure, along with visible swelling of the nanofibers (Fig. 4A).

At 14 days post-dressing application, the fibrous structure of the chitosan NF dressing was largely masked by accumulated exudate proteins and cells, making the structure difficult to distinguish. However, chitosan fibers remained discernible within the cracks of the removed and dried dressing, suggesting at least partial preservation of the fibrous structure in the wound environment (Fig. 4B–D).

### Re-epithelialization Process

**Fig. 5:**
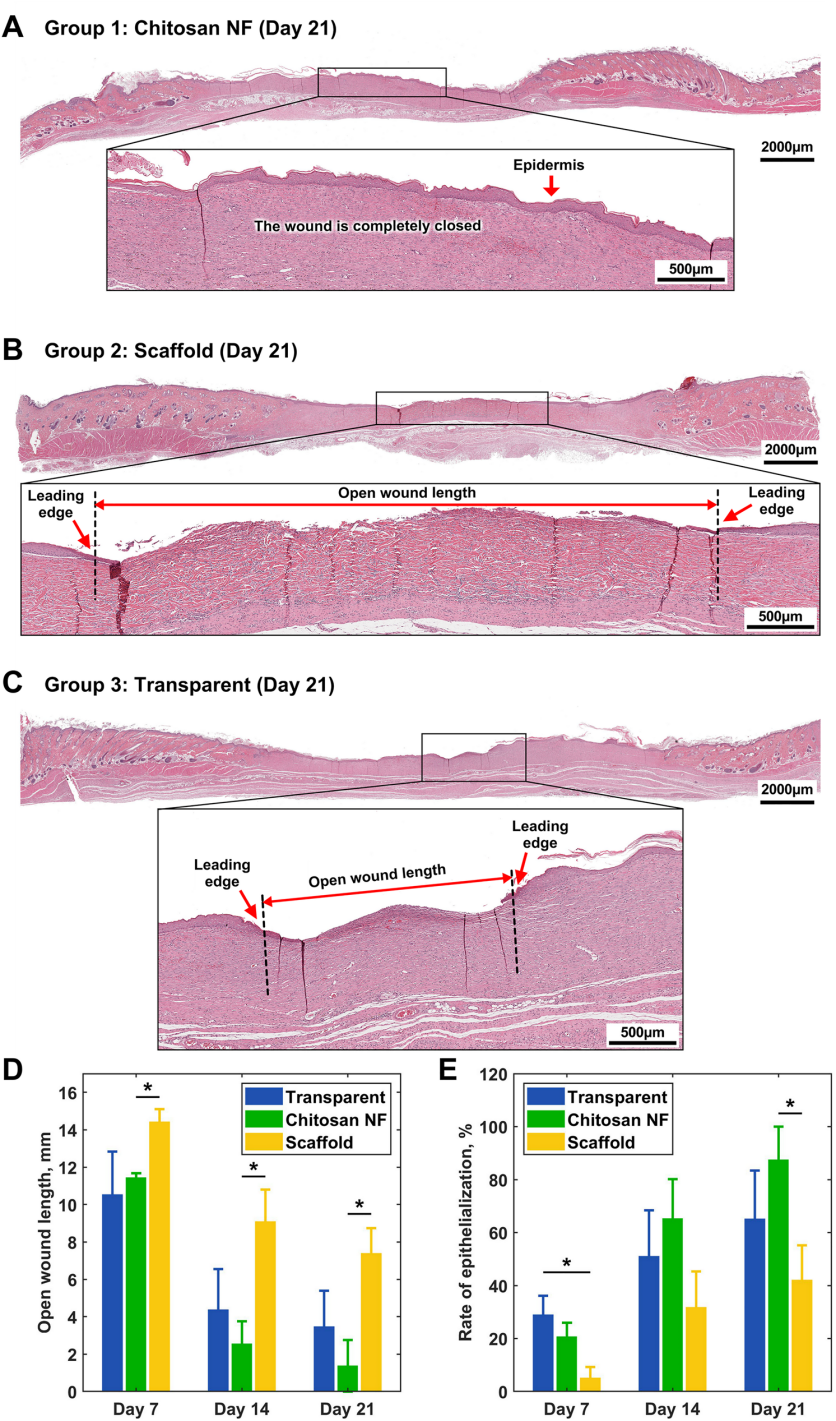
Assessment of the open wound length and rate of **e**pithelialization: **A**–**C** identification of open wound boundaries on H&E-stained histological sections of rats sacrificed 21 days post-wound infliction; **D** quantitative analysis of wound length across days 7, 14, and 21; **E** group-wise comparisons of the epithelialization rate. Data are represented as mean ± SEM; *p < 0.05

The newly formed epidermis was analyzed separately from the underlying granulation tissue to clearly delineate the epithelial border and visualize epithelial thickness, a key indicator of wound healing. Animals treated with scaffold dressing showed significantly larger open wound lengths compared to the chitosan NF group (Fig. 5D, p = 0.006, 0.021, 0.015 on days 7, 14, 21, respectively). Statistical comparisons between the chitosan NF and transparent dressing groups did not demonstrate any significant difference in open wound length throughout the study (Fig. 5C, D).

As illustrated in Fig. 5E, the re-epithelialization rate (%) observed on the 7th-day post-wounding was slower in the group treated with scaffold as opposed to those treated with transparent dressing (*p* = 0.02). There was no significant difference between chitosan NF and transparent dressing groups in this parameter. While the re-epithelialization rates on day 14 exhibited no substantial disparities among the groups, a significant acceleration was observed within the chitosan-treated group (*p* = 0.03) on day 21 when compared with the scaffold-treated group and no differences between the scaffold and transparent dressing groups.

**Fig. 6:**
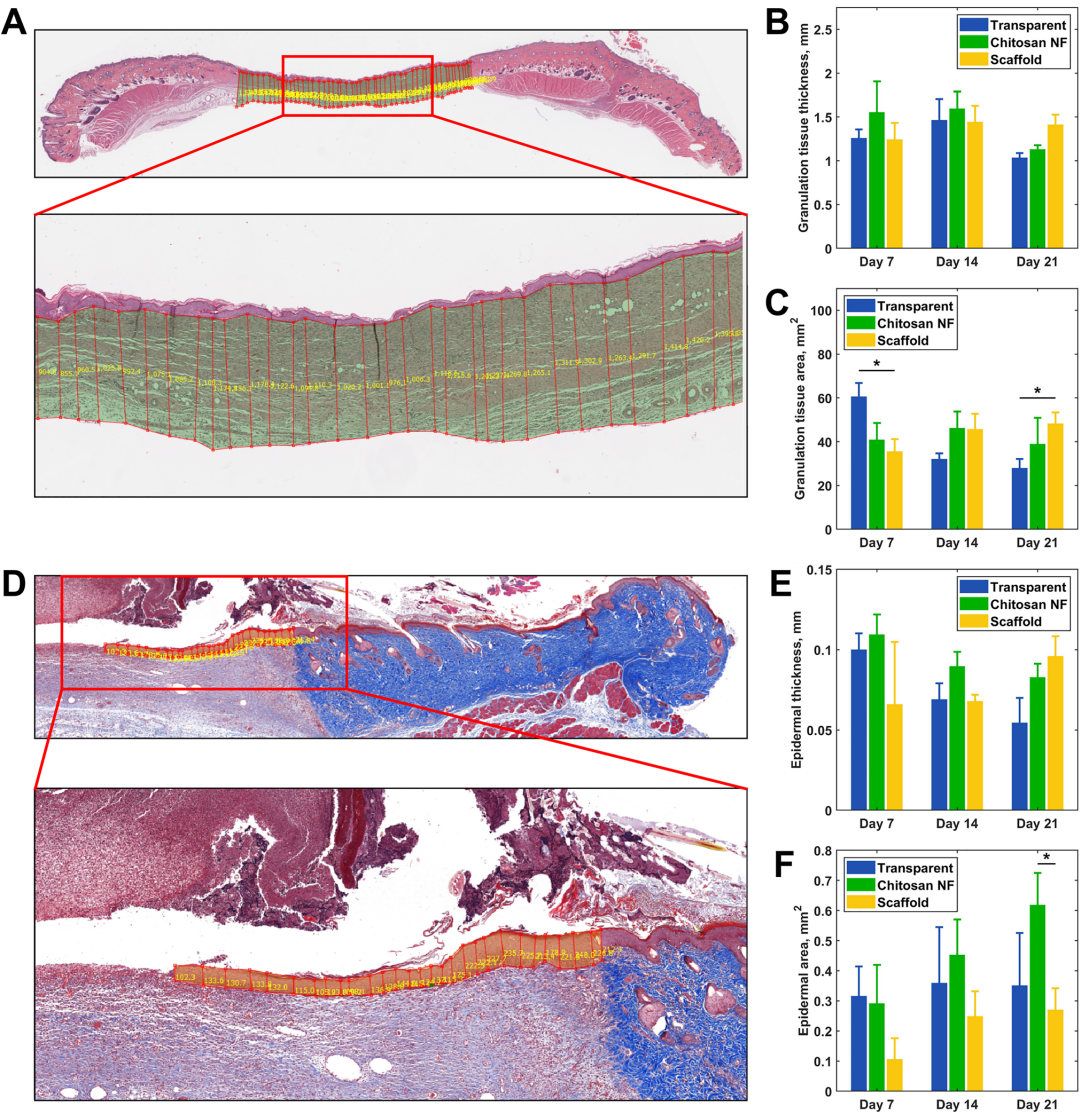
Evolution of granulation tissue and epidermal histological metrics: use of software to measure the thickness and cross-sectional area of the granulation tissue (**A**) and newly regenerated epidermis (**D**); a comparison of **B** granulation tissue thickness, **C** granulation tissue area, **E** epidermal thickness, and **F** epidermal area in rats with different dressings at days 7, 14, and 21. Group differences were assessed using an unpaired *t*-test. Data are presented as mean ± SEM; *p < 0.05

**Fig. 7:**
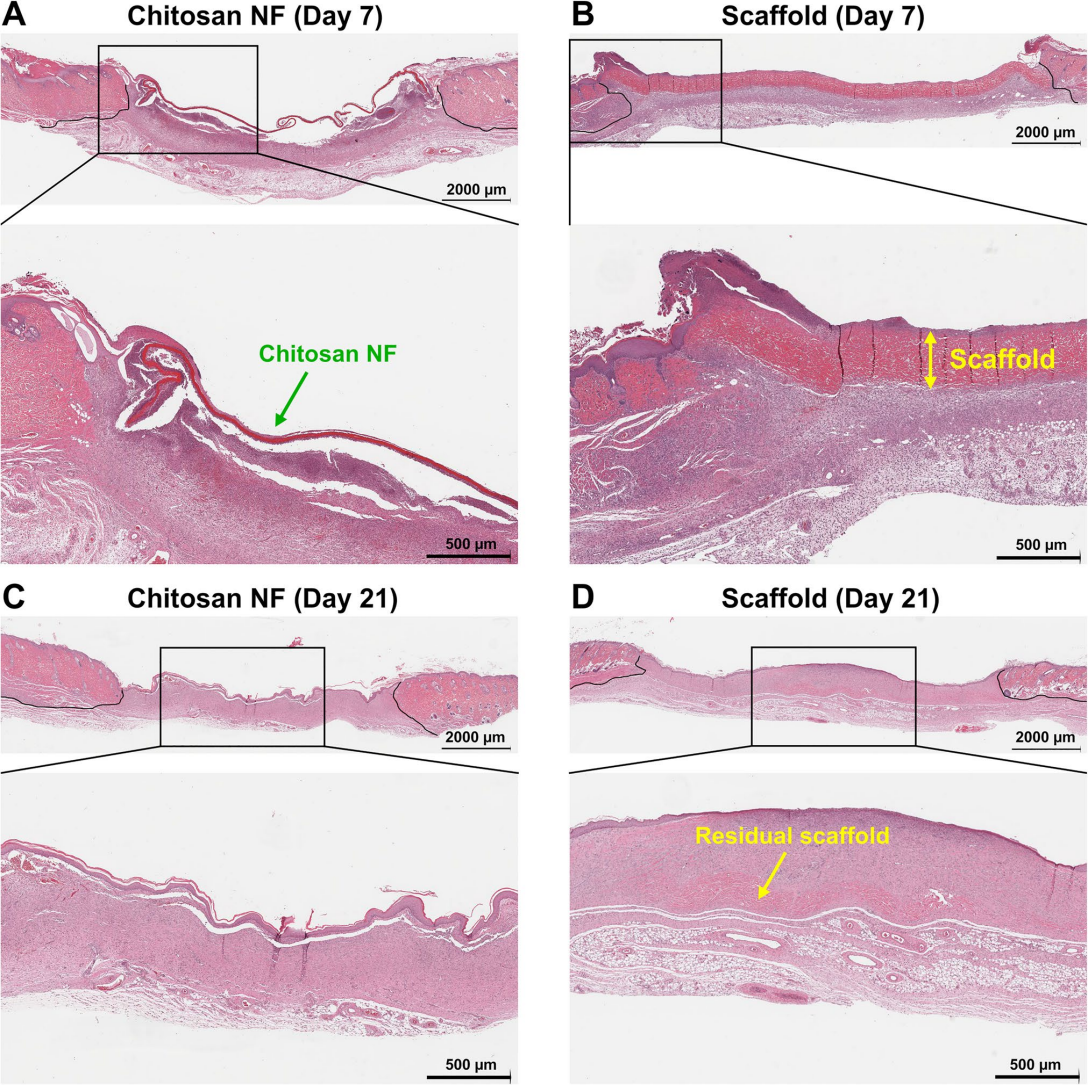
Histological cross sections of wound sites treated with chitosan NF and scaffold dressings at days 7 and 21 post-injury. **A** At day 7, chitosan NF remains visible within the wound bed (green arrow); **B** the commercial scaffold dressing appears largely intact at day 7 (yellow arrow); **C** by day 21, chitosan NF is largely degraded and no longer detectable; **D** in contrast, residual scaffold material is still present at day 21 (yellow arrow), indicating slower degradation; H&E staining

Further, on day 21 epidermal area evaluation revealed a pronounced enhancement (*p* = 0.03) in the new epidermal area in rats treated with chitosan NF dressing when contrasted against the scaffold group (Fig. 6F). The areas of granulation tissue in the group treated with transparent dressing exceeded those of the scaffold-treated group (60.5 ± 6.3 mm^2^ vs. 35.52 ± 5.6 mm^2^, *p* = 0.02) and exhibited no difference when compared to the chitosan group (*p* = 0.097) on day 7 (Fig. 6C). Conversely, on day 21, the granulation tissue area was larger in the scaffold group when contrasted against the transparent dressing group (48.2 ± 5.1 mm^2^ vs. 27.8 ± 4.2 mm^2^, *p* = 0.01). No differences between the groups were observed in granulation and epidermal tissue thicknesses (Fig. 6B and E). Figure 7 shows histological cross sections of wound sites treated with chitosan NF and commercial scaffold dressings, highlighting their positions within the wound bed.

### TNF-α and iNOS Concentrations in Wound Tissues

**Fig. 8:**
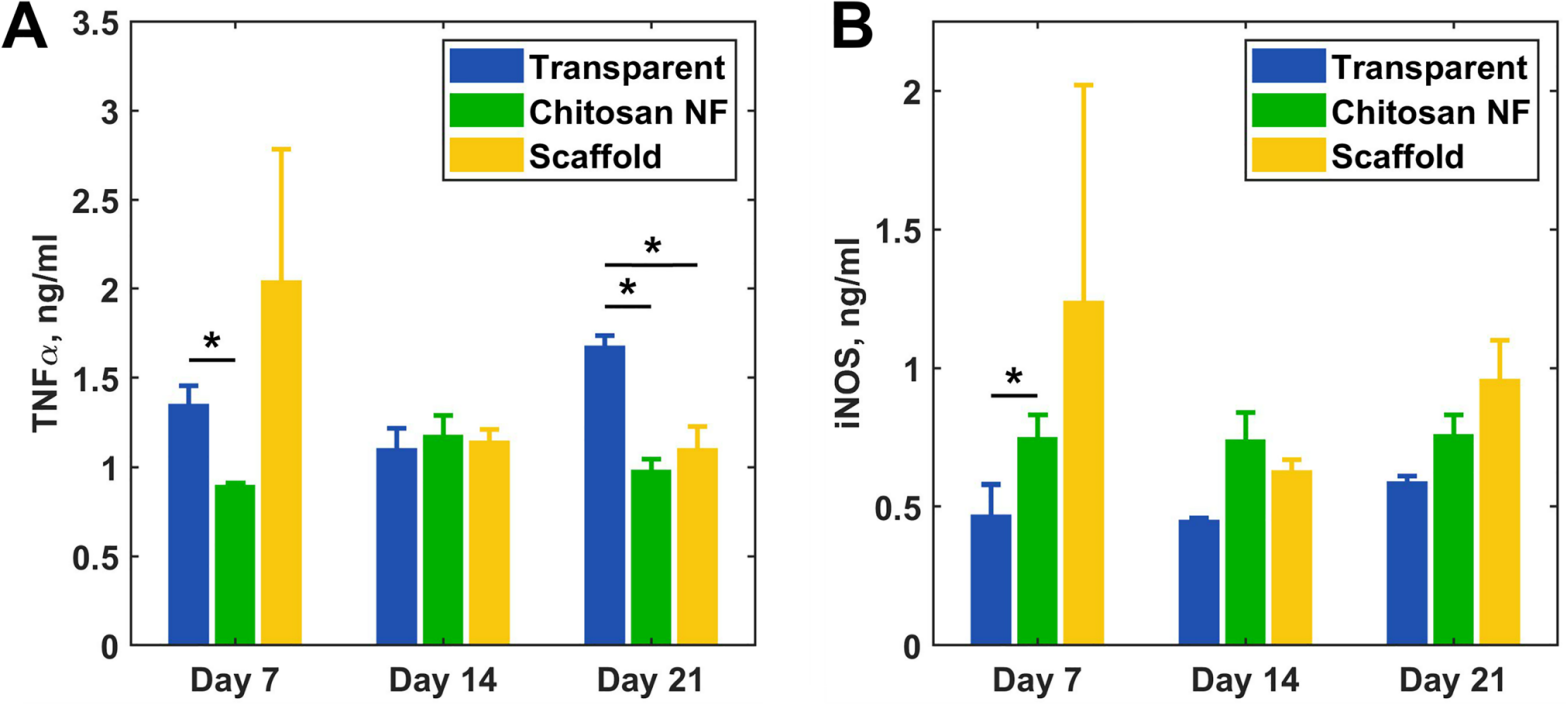
Evaluation of biological markers of wound healing in skin tissue biopsies: **A** Evolution of TNF-α concentration across time; **B** iNOS concentration variation over time. Values are represented as mean ± SEM; * *p*-value < 0.05

As illustrated in Fig. 8A, TNF-α protein levels in wound tissues on day 7 were significantly reduced in the chitosan-treated group compared to the transparent dressing group (*p* = 0.013). However, TNF-α concentrations in the scaffold-treated group did not show any significant difference from the transparent and chitosan groups (*p* = 0.445, and 0.259, respectively). By day 14, no discernible disparities in TNF-α levels were observed across all groups. On day 21, TNF-α concentrations were significantly reduced in the groups treated with chitosan NF dressing compared to the transparent dressing group, with concentrations of 983.2 ± 62.4 pg/ml versus 1677.7 ± 59.4 pg/ml, respectively (*p* < 0.001). A significant reduction was also observed in the scaffold group compared to the transparent dressing group, with concentrations of 827.8 ± 289.7 pg/ml versus 1677.7 ± 59.4 pg/ml, respectively (*p* = 0.028).

The level of iNOS enzyme was significantly higher in rats that received chitosan treatment compared to the transparent dressing group on day 7 (*p* = 0.026), while there were no differences in iNOS levels between the scaffold and transparent dressing groups during this period (*p* = 0.427). On days 14 and 21, no noticeable differences in iNOS levels were observed across all groups (Fig. 8B).

### Angiogenesis, Macrophage Density in the Granulation Tissue

**Fig. 9:**
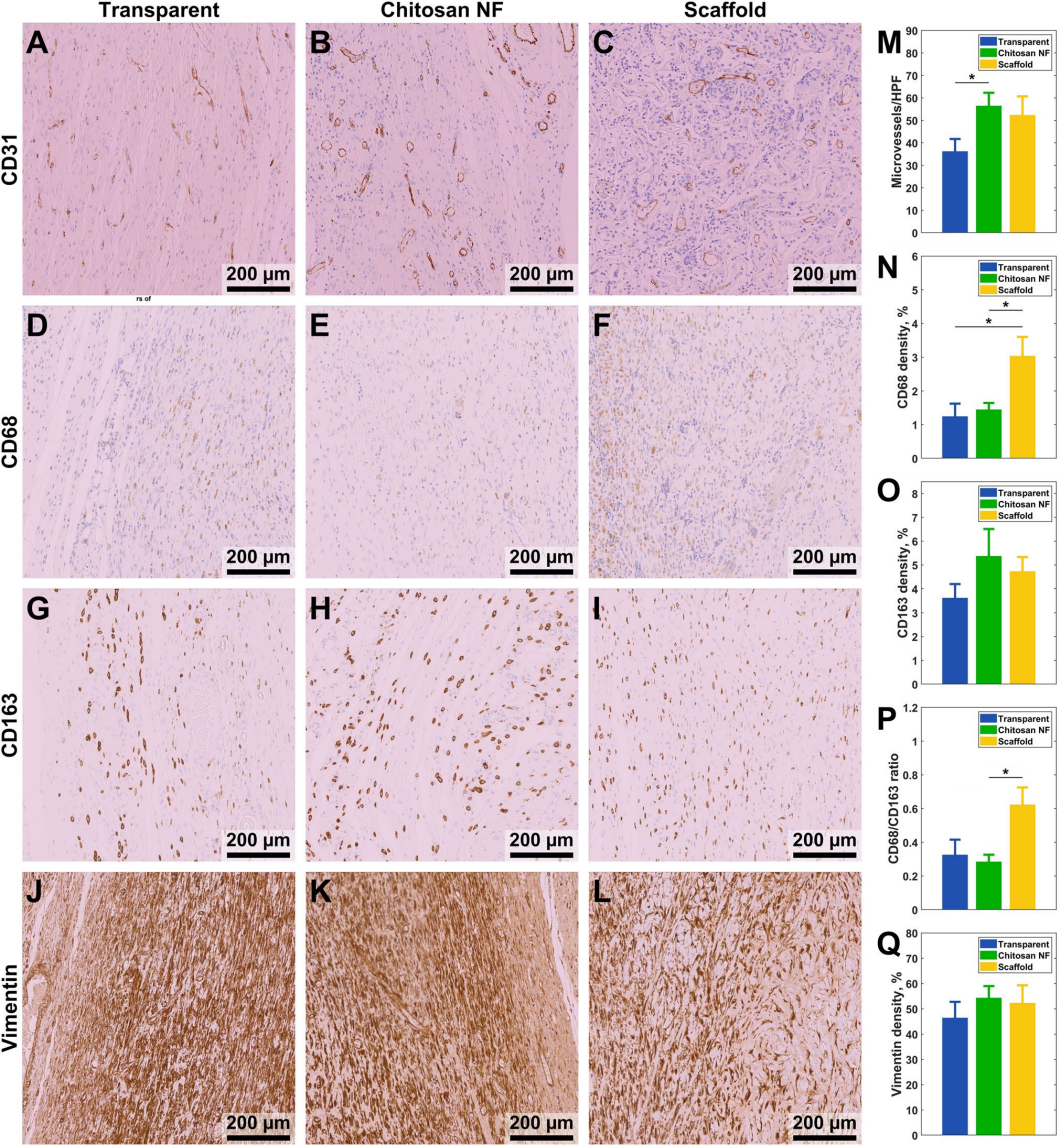
Immunohistochemistry staining of granulation tissue on day 21 after wounding. The histological scans are organized as follows: the left column is for transparent dressing, the middle column is for chitosan NF dressing, and the right column is for scaffold. **A**–**C** CD31^+^ staining highlights new blood vessels (endothelial cells in vessels shown in brown); **D**–**F** M1-type macrophages marked by CD68^+^ in the granulation tissue (in brown); **G**–**I** M2-type macrophages marked by CD163^+^ in the granulation tissue (in brown); **J**–**L** Vimentin filaments marked by anti-vimentin monoclonal antibody in the granulation tissue (in brown). The bar plots show **M** counts of new blood vessels; densities of **N** CD68^+^; **O** CD163^+^; **P** CD68^+^/CD163^+^ ratio; **Q** vimentin measured by color thresholding

The effect of dressing type on angiogenesis is demonstrated by CD31 immunostaining in Fig. 9A–C. The microvessel number in the granulation tissue was significantly higher (*p* = 0.045) in rats treated with chitosan NF dressing compared to those in the transparent dressing group on day 21 (Fig. 9M). There was no significant difference in the density of CD31 between rats that received the scaffold and those in the transparent dressing group.

Immunohistochemistry was used to investigate the density of M1 macrophages, which express the CD68^+^ marker, compared with M2 macrophages expressing CD163^+^. Figure 9D–F and G–I shows the density of macrophages with CD68^+^ and CD163^+^, respectively. There were no significant differences in the CD163^+^ among the three groups (Fig. 9O). However, the density of CD68^+^ in the scaffold group was significantly higher than in both chitosan NF (*p* = 0.037) and transparent dressing (*p* = 0.039) groups (Fig. 9N). Additionally, the CD68+/CD163+ ratio in the wound bed was significantly lower in rats treated with chitosan NF dressings compared to the scaffold group (*p* = 0.04) (Fig. 9P). However, no significant difference was observed between the transparent and scaffold groups (*p* = 0.07).

The expression of vimentin protein was evaluated by measuring its density in the granulation tissue (Fig. 9J–L). There were no significant differences in the density of vimentin protein among the three groups (Fig. 9Q).

### Qualitative Evaluation of Re-epithelialization and Epidermal Turnover

**Fig. 10:**
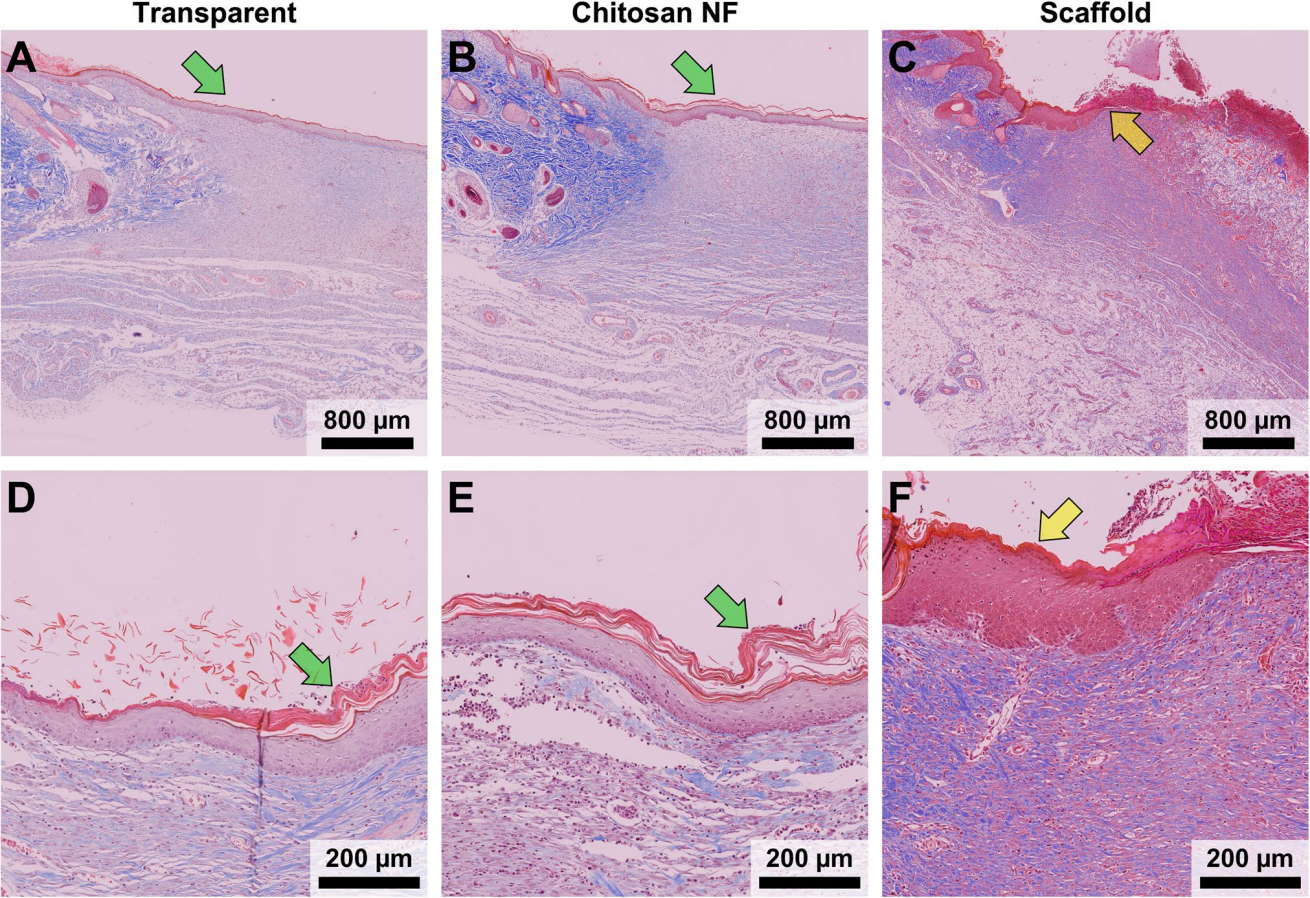
Qualitative assessment of epidermis formation progress. Green arrows in both the transparent dressing (**A**) and chitosan NF (**B**) groups indicate the newly formed epidermal layer, signifying that re-epithelialization was complete by day 21. In panel **C**, the yellow arrow highlights the leading edge of the epidermis layer, whose formation was significantly delayed in the scaffold group on day 21. Close-up photos of the transparent dressing (**D**) and chitosan NF **E** groups show green arrows pointing to the stratum corneum, which has separated from the underlying stratum granulosum, a process associated with normal epidermal turnover. In contrast, the epidermal layer in the scaffold group **F** shows reduced signs of turnover, as the cornified layer, indicated by the yellow arrow, remains closely attached to the granular layer

In both the chitosan NF and transparent dressing groups, re-epithelialization was nearly complete by the 21st day, marked by the presence of a newly formed epidermal layer (Fig. 10A, B). Conversely, in the scaffold group, this process was notably delayed by the same timeframe (Fig. 10C). Epidermal turnover, characterized by the continuous shedding of the stratum corneum and renewal of underlying layers, including the stratum granulosum which was evident in the chitosan NF and transparent dressing groups (Fig. 10D, E). In contrast, the epidermal layer in the scaffold group showed no distinct turnover, with the cornified layer remaining merged with the granular layer (Fig. 10F).

### Body Weight and Splint Integrity

In all three groups, the body weight loss did not exceed 4% of the initial weight. No significant differences in weight changes were observed between subgroups on days 7, 14, and 21. Throughout the study, all splints remained securely in place, with no instances of dislodgement observed, ensuring consistent conditions were maintained until the study’s conclusion. Additionally, the sutures remained intact and unchanged from their original placement.

## Discussion

The results of this study demonstrated that the application of chitosan NF dressing improves wound healing in a full-thickness acute wound model in rats. This favorable effect is related to the modulation of neovascularization, reduction of the inflammatory responses, and shifting in the pro-inflammatory to an anti-inflammatory macrophage state.

### Chitosan NF Dressing Enhances Re-epithelialization and Epidermal Turnover Over Scaffold Dressing, with Comparable Outcomes to Transparent Dressing

Complete re-epithelialization remains perhaps the most important outcome in numerous clinical wound healing evaluations [48]. In our study, rats treated with chitosan NF dressing exhibited re-epithelialization at a rate more than double that of rats treated with commercial bioactive scaffold. On day 21, the average area covered by the newly formed epidermis was 87.5% in the chitosan NF-treated group, compared to 65.4% in the transparent group and 42.0% in the scaffold group. Supporting our observations, a recent investigation by Movaffagh et al. underscored the potential of high molecular weight chitosan powder in accelerating epithelialization in full-thickness acute wounds of Wistar male rats [49]. Thuy et al. reported a significant increase in epidermal thickness using a chitosan hydrocolloid dressing on Sprague Dawley rats [16]. The underlying reasons for the increased re-epithelialization in wounds treated by chitosan-based dressings have not been thoroughly studied. However, Kitano et al. reported that iNOS-Knockout mice exhibited significant delays in granulation tissue formation and re-epithelialization in an excision wound model [50]. The increased level of iNOS protein in our chitosan-treated group may stimulate faster epithelialization compared to the scaffold group.

On the other hand, the reasons for inhibited epithelialization in rats subjected to commercial scaffold treatment, which lagged behind the chitosan NF and transparent dressing groups, also remain elusive. The investigation of the efficiency of the PriMatrix scaffold is largely confined to clinical trials [51, 52], with only one animal study in our best of knowledge, which involved subcutaneously implanting the scaffold in a mouse model and evaluating the remodeling of surrounding tissues [27]. One clinical investigation involving third-degree burns reported that, while engraftment and revascularization occurred on the wounds, discernible re-epithelialization was absent even 12 days after applying the scaffold to burns on the right leg [16]. Although some degree of re-epithelialization was observed in the left leg wound over time, this discrepancy highlights the potential moderate efficacy of PriMatrix in promoting re-epithelialization in acute full-thickness wounds.

The epidermal turnover time, the duration in which cells travel from the basal layer to the outer surface of the stratum corneum, is influenced by both the overall cell count and the number of newly generated cells [53]. The qualitative analysis demonstrated that epidermal turnover was significantly slower in the scaffold group compared to the other two groups. In the chitosan NF and transparent dressing groups, the cornified epidermis partially separated from the underlying living tissue, consistent with the typical healthy epidermal turnover cycle, which occurs approximately every 34 days in rats [54].

The similar wound length reduction observed between the chitosan NF and transparent dressing groups suggest that in this acute wound model, angiogenesis was not the primary rate-limiting factor for epithelial closure, as keratinocyte migration and proliferation over a minimally vascularized matrix [55] supported by the moist environment provided by Tegaderm were sufficient to achieve comparable re-epithelialization [56]. Prior studies have also demonstrated comparable wound closure rates for Tegaderm and Tegaderm combined with poly ɛ-caprolactone (PCL)/gelatin dressings [57]. In addition, similarly, Sparks et al. reported higher re-epithelialization and scab detachment scores with Tegaderm compared to an FDA-approved collagen scaffold (Integra, Integra LifeSciences, NJ) [58], highlighting Tegaderm’s advantage in acute wound healing.

### Chitosan NF Dressing Promotes Neovascularization and Cellular Proliferation Compared to Transparent Dressing

The results of this study suggest that the chitosan NF dressing enhances neovascularization compared to the transparent dressing group (Fig. 6M). Vascularization is essential for wound healing as it supplies nutrients and oxygen, sustaining cell viability and facilitating regeneration within skin wounds. In line with the findings of this study, chitosan hydrogel demonstrated its effectiveness in promoting angiogenesis [59]. The chitosan NF dressing accelerated the formation of new blood vessels, thereby enabling adequate delivery of oxygen and nutrients, which in turn supports the migration and proliferation of keratinocytes during wound healing.

The iNOS enzyme plays a critical role in activating the angiogenesis pathway, an essential process in wound repair. Its ability to enhance blood vessel formation underscores its importance as a key parameter in our analysis [60]. Our data indicated that by day 7 post-wounding, chitosan NF dressing significantly increased iNOS concentrations in wound tissues compared to the transparent dressing. According to Chin et al., iNOS plays a crucial role in angiogenesis signaling during the first week of skin wound healing [61]. Early in the healing process, macrophages generate iNOS, which is pivotal for cellular proliferation and collagen formation [62]. However, at later time points (days 14 and 21), the iNOS levels were consistent across all groups. Supporting our findings, fr et al. reported that iNOS gene expression is heightened during the initial and later inflammatory phases of wound repair, typically peaking around days 1–7 [64].

### Chitosan NF Dressing Enhances Anti-inflammatory Response

Immunohistochemical examinations of the granulation tissue showed that the density of CD68^+^ macrophages, indicative of pro-inflammatory activity, was higher in rats that received scaffold dressing compared to those treated with chitosan NF and transparent dressings. In contrast, the density of anti-inflammatory CD163^+^ macrophages showed no significant difference among the groups. Consequently, the CD68^+^/CD163^+^ macrophage ratio was significantly lower in rats treated with chitosan NF compared to the scaffold group, indicating a shift toward a more anti-inflammatory environment.

Macrophage polarization in skin wound healing typically transitions from M1 (pro-inflammatory) to M2 (anti-inflammatory), with the M1/M2 ratio serving as a potential indicator of wound age [63]. CD68^+^ is predominantly expressed by M1 macrophages, which release cytokines like TNF-α, IL-1β, and IL-6, contributing to inflammation. Conversely, CD163^+^ is primarily expressed by M2 macrophages [42]. A decrease in the CD68^+^/CD163^+^ ratio suggests a relative increase in M2 macrophages compared to M1 macrophages within the wound microenvironment. This shift likely accounts for the observed lower TNF-α protein levels on day 21 in the groups treated with chitosan NF dressing, reflecting a transition from a predominantly pro-inflammatory to an anti-inflammatory state.

Interestingly, while the CD68 density and CD68^+^/CD163^+^ macrophage ratio are higher in the scaffold group, the level of TNF-α is lower in this group compared to the transparent dressing group. Although macrophages are typically the primary producers of TNF-α [65], we did not find any reports addressing the observation of lower TNF-α levels alongside higher CD68^+^ marker levels in a wound healing assessment.

However, CD68 is not exclusively a marker for M1 macrophages. It is also expressed by M2 macrophages, as well as fibroblasts and endothelial cells [66, 67]. Therefore, the elevated CD68^+^ expression in the scaffold group may reflect an increased presence of various cell types, including M2 macrophages, fibroblasts, and endothelial cells, which collectively contribute to a reparative wound environment.

In contrast, the TNF-α protein level was significantly higher in the transparent dressing group on day 21, highlighting its role as a key pro-inflammatory cytokine critical in the acute inflammatory response to tissue damage, primarily produced by immune cells like macrophages and neutrophils [68]. TNF-α plays a critical role in wound healing by regulating a wide range of biological functions. The non-linear pattern of TNF-α over time aligns with its established role in wound healing, where TNF-α peaking during the early inflammatory phase [69], followed by a decline and a fluctuating trend as healing progresses [70, 71].

Notably, it is known to inhibit several key cellular processes essential for tissue repair, including fibroblast proliferation, collagen synthesis, and keratinocyte migration [72]. Therefore, reducing TNF-α levels can alleviate these inhibitions, promoting cell proliferation, migration, and collagen organization [75, 77]. The chitosan NF dressing was associated with a low immunogenic response, as evidenced by reduced TNF-α expression during the early inflammatory phase and a lower M1/M2 macrophage ratio, indicating a shift toward a pro-healing immune environment.

The anti-inflammatory activity of chitosan nanofibrous dressings, leading to reduced TNF-α expression, may stem from a decrease in macrophage counts, with these cells being the predominant producers of TNF-α [79]. The structural features of nanofibrous dressings, such as their surface topography and porosity, may positively influence macrophage behavior during the healing process [80]. Furthermore, Chang et al. noted that high molecular weight chitosan can suppress pro-inflammatory cytokines like TNF-α and IL-6 through lipopolysaccharide mediation [81].

### Potential Mechanisms of Chitosan in Enhancing Acute Wound Healing

**Fig. 11:**
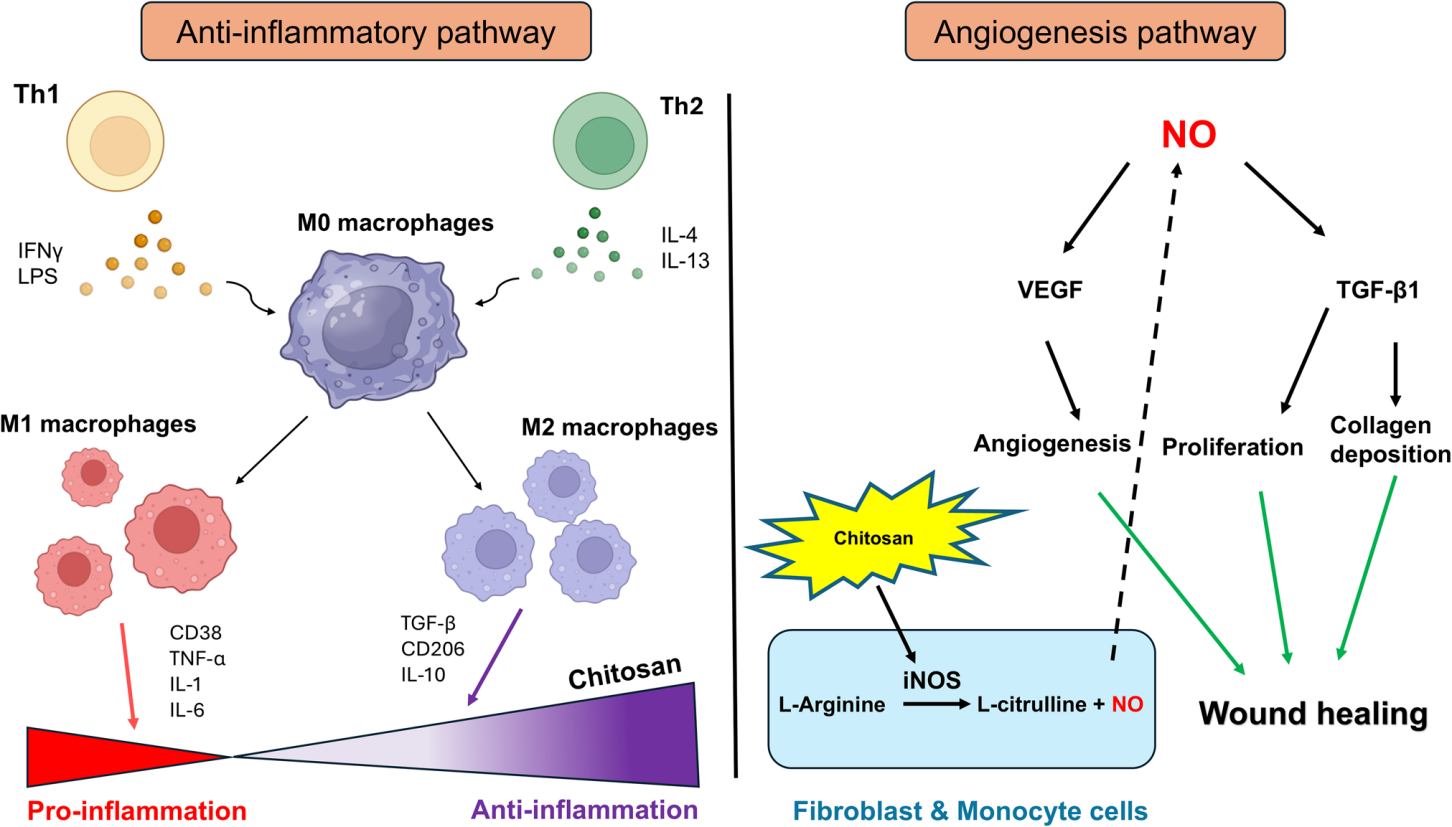
Possible mechanisms by which chitosan improves acute wound healing in rats. The left panel illustrates the anti-inflammatory pathway, showing how chitosan reduces inflammation in the wound tissues. The right panel depicts the proposed mechanism by which chitosan enhances angiogenesis leading to improved wound healing

Figure 11 illustrates the potential mechanisms for the anti-inflammatory response and angiogenesis in chitosan-treated wounds. In the inflammatory response, Type 1 T helper cells (Th1) can release agents such as bacterial lipopolysaccharides (LPS) and interferon-gamma (IFN-γ), which promote the polarization of macrophages toward M1. Conversely, Th2 cells can induce polarization toward M2 macrophages by releasing anti-inflammatory cytokines like interleukin (IL)-4 or IL-13. By day 21, in the chitosan NF group, the level of TNF-α had decreased compared to the transparent dressing group, and the CD68^+^/CD163^+^ ratio was also lower than in the scaffold group.

The proposed mechanism by which chitosan promotes angiogenesis involves an increase in iNOS levels in the chitosan-treated group compared to the control groups. This enzyme stimulates the production of nitric oxide (NO), a gaseous messenger that enhances angiogenesis through vascular endothelial growth factor (VEGF) [73]. As a result, angiogenesis is elevated in the chitosan group compared to the control group.

### Study Limitations

This study employed young Sprague Dawley rats as model organisms, which may not accurately mimic typical human wound conditions due to inherent physiological differences between species. Furthermore, the study focused on young animals, thus overlooking the variations expected in a population that includes both young and aged individuals. The acute wound model in these healthy animals fails to capture chronic comorbidities like diabetes, vascular diseases, and immune system disorders, which commonly affect wound healing in humans. A more comprehensive comparative assessment might require including a wider range of dressing types to fully evaluate the healing capabilities of chitosan NF dressing in diverse clinical scenarios.

While splinting proved effective in inhibiting wound contraction, it did not completely eliminate it in certain cases. To address this limitation, future studies should explore strategies to fully prevent wound contraction, such as optimizing suturing techniques or refining the design and shape of the splint.

Another limitation of this study is the lack of a true negative control group (i.e., untreated wounds), which would have provided a clearer reference point for the natural baseline healing process. While Tegaderm was employed as a clinically relevant dressing to prevent infection and desiccation [74], future studies should incorporate an untreated control group to enhance the mechanistic interpretation of the findings.

In this study, open wound length was selected as the primary metric for wound healing assessment due to its reproducibility and widespread acceptance in similar research [76]. This approach minimizes variability associated with irregular wound shapes and provides a straightforward method for analysis [82]. However, the inability to clearly distinguish the open wound surface from newly formed epithelium in top-view images, caused by similar coloring, prevented the measurement of wound area. This limitation is significant, as wound area provides a more comprehensive parameter for evaluating healing progress. Future studies should prioritize incorporating precise wound area measurements to achieve a more holistic and detailed evaluation of the healing process.

This study highlighted the beneficial effects of chitosan nanofibrous wound dressing compared to commercial modern dressings on wound healing in an acute wound model in rats. These effects were linked to the restoration of the anti-inflammatory response and an increase in iNOS content, and angiogenesis. Moreover, the observed transition from an inflammatory to an anti-inflammatory wound environment under the chitosan NF treatment is related to a decrease in the M1/M2 macrophage ratio. Importantly, while chitosan NF improved epithelialization compared to the scaffold dressing, it demonstrated comparable performance to the polyurethane film (Tegaderm) in promoting re-epithelialization.

These findings suggest that the chitosan NF dressing has the potential to serve as a cost-effective therapy for impaired wound healing. A kilogram of medical-grade chitosan—sufficient to produce approximately 80 m^2^ of dressing—costs only a few hundred USD and can be processed in a single step using standard industrial equipment. This makes it a highly economical alternative to living tissue-derived dermal repair materials, which can cost thousands of dollars per square inch [78]. Nevertheless, further validation through preclinical studies remains essential for confirming its safety, efficacy, and clinical potential.

## Supplementary Information

Below is the link to the electronic supplementary material.

Supplementary file1 (PDF 976 kb)

## Data Availability

The datasets used or analyzed during the current study are available from the corresponding author upon reasonable request.
