# Supplementary material for: Chitosan Nanofibrous Dressing Increased Angiogenesis and Anti-inflammatory Response in an Acute Wound Model in Rats: A Comparative Study
Source: Ann Biomed Eng. 2025 Oct 8;53(12):3415–34. doi: 10.1007/s10439-025-03842-8 (PMC12686052; doi:10.1007/s10439-025-03842-8)
Supplement: Supplementary file 1 — Supplementary file1 (PDF 976 kb) [file 10439_2025_3842_MOESM1_ESM.pdf]

SUPPLEMENTARY INFORMATION

ARRIVE Essential 10

1. STUDY DESIGN

Study design: This study utilized Sprague Dawley rats, 3 months old, divided into three groups based on the type of wound dressing applied:

- Group 1: Control - wounds were dressed solely with Tegaderm (3M).
- Group 2: Conventional therapy - wounds were dressed with PriMatrix Dermal Repair Scaffold (Integra LifeSciences) and covered by Tegaderm.
- Group 3: Nanofiber - wounds were dressed with a chitosan nanofibrous wound dressing and covered by Tegaderm.

On Day 0, a full-thickness dermal wound measuring 2 cm x 2 cm was created on the dorsal area of each animal. A wound splint was then sutured to the wound edges to prevent contraction, and the wound was covered with a dressing.
In each group, rats were sacrificed at intermediate (7 and 14 days; 4 rats each) and final (21 days; 5 rats) time points.
The experimental unit: a single animal.

2. SAMPLE SIZE

Following data collection, a post-hoc sample size calculation was conducted using the collected data to determine the necessary statistical power. The primary endpoint was the open wound length. Based on the observed mean differences between the chitosan NF dressing group and the transparent group, we calculated the required sample size (N) for each group using the formula:

$$\text{n} = \frac{(z_{1-\alpha/2}+z_{1-\beta})^2 \times (s_1^2+s_2^2)}{d^2},$$

where $d^2 = (\mu_1 - \mu_2)^2$ represents the squared difference between the means of the two groups, where μ_1 and μ_2 are the means and s_1^2 and s_2^2 are the variances (standard deviations squared) of the experimental and control groups, respectively. $z_{1-\alpha/2}$ is the critical value for the chosen alpha level (1.96 for $\alpha = 0.05$), and $z_{1-\beta}$ is the critical value for the desired power (0.84 for 80% power). The minimum number of animals required per group for each time point is as follows:

Day 7: $n = \frac{(1.96+\ .84)^2 \times (1.34^2+0.49^2)}{(14.43-11.43)^2} = 1.77;$

Day 14: $n = \frac{(1.96+0.84)^2 \times (3.44+2.41^2)}{(9.08-2.54)^2} = 3.23;$

Day 21: $n = \frac{(1.96+0.84)^2 \times (3.05^2+3.07^2)}{(7.38-1.37)^2} = 4.06;$

In our study, we used four animals per group for the Day 7 and Day 14 time points, and five animals for the Day 21 time point.

3. INCLUSION AND EXCLUSION CRITERIA

Inclusion criteria: Rats included in this study were 3 months old and confirmed to be in good health before the experiment.
Exclusion criteria: The animals were excluded if the wound creation process resulted in excessive bleeding (determined by substantial blood loss during the procedure), if the wound edges were irregular, or if the wound size did not meet the specified dimensions. Additionally, rats were excluded if they showed signs of infection or died prematurely, thereby preventing the collection of histological and biological data.

	Group 1	Group 2	Group 3
Number of rats allocated to this study	13	13	13
Number of rats that died prematurely	0	0	0
Number of rats excluded for excessive bleeding	0	0	0
Number of rats excluded for irregular wound size	0	0	0
Number of rats excluded for infection	0	0	0

4. RANDOMIZATION

Randomization: The experimental units were randomly allocated to control and treatment groups. The randomization was performed using the standard *randperm* function in MATLAB for random permutation of integers.

Potential confounders: Each unit received a single treatment. All measurements were made after sacrifice within the same timeframe for biopsy fixation and freezing. All animals were single-housed in the same room after treatment. However, the location of cages on the shelves was not controlled.

5. BLINDING

During group allocation, outcome assessment, and data analysis, animals and biopsy specimens were marked with digital codes to maintain blinding. However, blinding was not employed during the experimental procedures due to the visual differences between the dressings and the specialized handling they required. This made it impractical for personnel involved in applying the dressings and monitoring the animals post-surgery.

6. OUTCOME MEASURES

Primary outcome measure: open wound length.
Outcome measures: average thicknesses and areas of the epidermis and granulation tissue, rate of epithelialization, density of new blood vessels marked by CD31, vimentin density, CD68+ and CD163+ macrophages marker density.

7. STATISTICAL METHODS

Statistical analysis was conducted using IBM SPSS software (Version 29). An unpaired t-test was employed to assess the rate of epithelialization, wound length, epidermal thickness, epidermal area, granulation tissue thickness, granulation tissue area, number of vessels, density of CD68+ and CD163+ markers, and iNOS protein levels between the groups. One-way analysis of variance (ANOVA) followed by Tukey's HSD post-hoc test was used to evaluate the TNF-α concentration. All results are expressed as mean ± standard error of the mean (SEM), and differences were considered statistically significant with two-tailed p-values < 0.05.

8. EXPERIMENTAL ANIMALS

Male and female Sprague Dawley rats, approximately 3 months old, were used in the experiments. The animals were sourced from Charles River Laboratories.

9. EXPERIMENTAL PROCEDURES

The animals were acclimatized for one week after their arrival at the animal facility.
On day 0, skin wounding was performed on the dorsal part of the rat skin under general inhalational isoflurane anesthesia. The animal was kept on a warming pad during surgery and maintained in a prone position. Adequate sedation was ensured with a toe or tail pinch. Buprenorphine HCL SR at a dose of 1.0 mg/kg (0.15 ml) was administered subcutaneously into the left hind leg. The dorsum was clipped circumferentially around the chest. A 2 cm x 2 cm square was marked out just below the inferior borders of the scapula in the center of the dorsal surface. The subject was then prepped and draped in the standard fashion.

Using a No. 15 scalpel blade, a 2 cm x 2 cm square incision was made along the marking through the skin and dermis. The full-thickness square was then easily elevated with forceps and removed from the back using dissecting scissors. A frame-shaped square splint (2 cm x 2 cm inner cutout dimensions, aluminum 0.025" thick) was sutured over the skin surrounding the wound using non-absorbable 5-0 nylon sutures to prevent wound contracture. The wound was then dressed according to the group allocation of the test subject:

- Group 1: The wound bed, splint, and surrounding area were covered with Tegaderm.
- Group 2: The wound bed was dressed with a 2 cm x 2 cm PriMatrix. Then the dressed wound, splint, and surrounding area were covered with Tegaderm.
- Group 3: The wound bed was dressed with a 2 cm x 2 cm chitosan nanofibrous wound dressing. Then the dressed wound, splint, and surrounding area were covered with Tegaderm.

After the application of the dressing, the thorax was wrapped circumferentially with a 1-inch-wide stretch cotton gauze, encircling the thorax about two times. The gauze was applied with just enough stretch to ensure it did not slip off. No compression or constriction was applied with this wrap to allow for normal respiration. The natural increase in girth from the thorax to the abdomen tends to keep such a circumferential thorax dressing in place. After the cotton gauze was applied, the thorax was wrapped without tension using Elastikon adhesive bandage tape. No compression or constriction was applied with this wrap to allow for normal respiration.

Digital images were acquired of the wound area every 2-3 days, and at each time point of 7, 14, and 21 days. When taking the digital images, the wound protective wrappings were removed, but the wound treatments, i.e., Tegaderm, PriMatrix, or the chitosan nanofibrous wound dressing, were not disturbed. The animals were placed under brief

sedation with inhaled isoflurane administered through a nose cone, allowing the wound protective wrappings to be removed, pictures to be taken, and new wrappings applied.

10. RESULTS

Summary/descriptive statistics for the experimental groups (mean ± standard error):

Outcome measure	Units	Group 1			Group 2			Group 3		
		Day 7	Day 14	Day 21	Day 7	Day 14	Day 21	Day 7	Day 14	Day 21
Open wound length	mm	10.5±2.3	4.37±2.18	3.47±1.91	14.4±0.67	9.09±1.72	7.38±1.36	11.4±0.25	2.55±1.21	1.37±1.37
Rate of epithelialization	%	28.9±7.2	51.0±17.4	65.2±18.2	5.1±4.2	31.7±13.6	42.0±13.1	20.7±5.3	65.3±14.9	87.5±12.5
Granulation tissue thickness	mm	1.26±0.10	1.46±0.24	1.03±0.05	1.24±0.19	1.44±0.18	1.41±0.11	1.55±0.35	1.59±0.20	1.13±0.05
Granulation tissue area	mm^2	60.5±6.3	31.9±2.8	27.8±4.2	35.5±5.6	45.6±7.1	48.3±5.1	40.8±7.7	46.1±7.7	38.9±12.0
Epidermal thickness	µm	100.0±10.2	67.0±10.1	54.4±15.5	65.9±38.9	67.8±4.15	95.9±12.4	109.3±12.6	89.6±9.1	82.7±8.6
Epidermal area	mm^2	0.316±0.98	0.359±0.186	0.351±0.175	0.106±0.70	0.248±0.84	0.270±0.72	0.291±0.129	0.452±0.118	0.618±0.107
TNF-α concentration	ng/ml	1.35±0.11	1.10±0.12	1.68±0.06	2.05±0.74	1.15±0.07	1.10±0.12	0.90±0.01	1.18±0.11	0.98±0.06
iNOS concentration	ng/ml	0.47±0.11	0.45±0.01	0.59±0.02	1.24±0.78	0.63±0.04	0.96±0.14	0.75±0.08	0.74±0.10	0.76±0.07
Microvessels/HPF	counts	n/a	n/a	36.2±5.5	n/a	n/a	52.3±8.4	n/a	n/a	56.5±5.8
CD68 density	%	n/a	n/a	1.24±0.38	n/a	n/a	3.04±0.56	n/a	n/a	1.45±0.20
CD163 density	%	n/a	n/a	3.62±0.57	n/a	n/a	4.73±0.60	n/a	n/a	5.37±1.14
CD68/CD163 density ratio	-	n/a	n/a	0.33±0.09	n/a	n/a	0.62±0.10	n/a	n/a	0.29±0.04
Vimentin density	%	n/a	n/a	46.4±6.4	n/a	n/a	52.3±7.0	n/a	n/a	54.3±4.7
